# Trajectory Optimization to Enhance Observability for Bearing-Only Target Localization and Sensor Bias Calibration

**DOI:** 10.3390/biomimetics9090510

**Published:** 2024-08-23

**Authors:** Jicheng Peng, Qianshuai Wang, Bingyu Jin, Yong Zhang, Kelin Lu

**Affiliations:** 1 School of Automation, Southeast University, Nanjing 210096, China; pengjicheng@seu.edu.cn (J.P.); xiaoshuaiwang703@gmail.com (Q.W.); 2 School of Automation Engineering, Nanjing University of Aeronautics and Astronautics, Nanjing 210016, China; chainajby94@nuaa.edu.cn; 3 Unmanned Aerial Vehicles Research Institute, Nanjing University of Aeronautics and Astronautics, Nanjing 210016, China; yongzhang@nuaa.edu.cn

**Keywords:** trajectory optimization, target localization, observability enhancement, control barrier function, bio-inspiration

## Abstract

This study addresses the challenge of bearing-only target localization with sensor bias contamination. To enhance the system’s observability, inspired by plant phototropism, we propose a control barrier function (CBF)-based method for UAV motion planning. The rank criterion provides only qualitative observability results. We employ the condition number for a quantitative analysis, identifying key influencing factors. After that, a multi-objective, nonlinear optimization problem for UAV trajectory planning is formulated and solved using the proposed Nonlinear Constrained Multi-Objective Gray Wolf Optimization Algorithm (NCMOGWOA). Simulations validate our approach, showing a threefold reduction in the condition number, significantly enhancing observability. The algorithm outperforms others in terms of localization accuracy and convergence, achieving the lowest Generational Distance (GD) (7.3442) and Inverted Generational Distance (IGD) (8.4577) metrics. Additionally, we explore the effects of the CBF attenuation rates and initial flight path angles.

## 1. Introduction

In recent years, the popularity of Unmanned Aerial Vehicles (UAVs) has increased because of their affordability and versatility [1,2,3]. These characteristics have facilitated the utilization of UAVs across multiple domains and applications, including infrastructure inspection [4], environmental monitoring [5], rescue missions and research [6], mapping [7], surveillance [8], and remote sensing [9]. In these sensing applications, target localization plays a critical role, which involves determining the position of a target via relative information between the UAV and the target, obtained from airborne sensors equipped on UAVs [10]. However, target localization based on passive sensors faces two main challenges. The first is that the bearing measurement bias introduces substantial errors in target localization [11]. The second challenge is that the estimation accuracy may be reduced because of the reliance solely on bearing information [12].

Target localization, which is usually aimed at obtaining a target’s inertial position, necessitates obtaining bearing information relative to the world frame [13,14]. Consequently, attitude sensors and onboard cameras, which provide the UAV’s attitude and the target’s line of sight (LOS) angle relative to the UAV’s fuselage frame, respectively, are generally integrated into the bearing-only target localization problem. According to references [15,16], targets can be accurately localized when the bearing measurements are perturbed only by Gaussian noise. However, in practice, this assumption is not valid. The outputs from both the attitude sensor and the onboard camera are biased [17]. Neglecting the bias can notably impair the target localization performance [11,17]. Considerable research efforts have focused on calibrating sensor biases. The method proposed in [18] constructs the bias pseudo measurement exclusively through the manipulation of local tracks, covariances, and the equivalent bias measurement matrices to estimate the sensor bias in sensor registration. The authors of [19] integrated sensor calibration and trajectory fusion within a multi-target tracking framework to mitigate the effect of bearing measurement bias on target tracking. The integration of data is crucial, particularly when a target needs to be observed simultaneously by two radar devices. In terms of state estimation, the EKF is a useful method for dealing with diffuse white noise models [20]. It also has the advantage of high computational efficiency compared with the Unscented Kalman Filter (UKF), which has been widely used in recent years for nonlinear filtering problems [21]. Given that the observability of the system directly impacts the performance of the state estimation [22,23], it is necessary to maintain and enhance the observability of the target localization system.

Observability is a fundamental property of a system that indicates its ability to uniquely determine an initial state from its outputs. Only if the system is observable can the states at any time be determined by a state estimator such as the Kalman Filter. The Fisher Information Matrix (FIM) is commonly used as a metric for assessing system observability [24,25]. The inverse of FIM corresponds to the Cramer–Rao Lower Bound (CRLB), which sets a theoretical lower limit on the covariance matrix of an ideal estimator, thereby representing the best achievable performance in state estimation. As highlighted in [12], maximizing system observability typically involves maximizing the determinant of the FIM, which in turn minimizes the estimation error covariance of the filter, enhancing its overall performance.

The observability of a bearing-only measurement system is dynamic and depends on the relative positions of the observer and the target [26,27,28,29,30]. Therefore, it is necessary to enhance the system’s observability by trajectory optimization. The authors of [31] utilized the rank of the observability matrix as a criterion to assess the system’s observability and to determine the reliability of different sensor locations. In [24,32,33], the determinant of the FIM was employed as a metric. It is maximized to generate the optimal trajectories that enhance the system’s observability. Unfortunately, there has been no investigation in the literature into UAV trajectory optimization for target localization based on directly enhancing system observability.

This work first analyzes the observability of a target localization system with biased bearing measurements via the Lie derivative method. It derives the conditions necessary to maintain system observability. To ensure observability, inspired by the phototropism of plants, a control barrier function was designed. This function restricts UAV motion, allowing it to avoid areas that may affect observability, with adjustable avoidance levels. Additionally, the condition number of the system observability matrix was employed as a metric to quantify the system observability, helping to identify the factors that contribute to system observability. Based on this analysis, a multi-objective, nonlinear programming problem was established to maintain and enhance system observability. To effectively solve the multi-objective, nonlinear programming problem, a penalty function was integrated into the Multi-Objective Gray Wolf Optimization Algorithm to address nonlinear constraints. Simulations confirmed the effectiveness of the proposed method. The UAV operated at a fixed altitude, modeled on a 2D, obstacle-free map, with constraints on the speed and turn rate of the UAV to limit its turning radius. The root-mean-square error (RMSE) of localization was used as a performance metric indicator for localization accuracy. The effectiveness of the proposed Nonlinear Constrained Multi-Objective Gray Wolf Optimization Algorithm (NCMOGWOA) was verified through comparisons with the Multi-Objective Particle Swarm Optimization Algorithm (MOPSOA) [34], Multi-Objective Arithmetic Optimization Algorithm (MOAOA) [35], and Sequential Quadratic Programming (SQP) method [36]. While the MOPSOA and MOAOA are both heuristic and neglect the fitness among the nondominated solutions, the SQP method requires a suitable starting point. To address these limitations, we propose the NCMOGWOA, which shows faster convergence and lower localization error in simulations. It outperforms the other methods in terms of the convergence metrics GD and IGD. Additionally, we explore the impact of the CBF attenuation rates and initial flight path angles on trajectory optimization.

The main contributions of this paper include the following:

(1) Observability Analysis: Deriving necessary conditions for maintaining the observability of the target localization system using only biased bearing measurements. A control barrier function is designed to ensure system observability by restricting UAV motion.
(2) Optimization Metric: Utilizing the condition number of the observability matrix as a metric. A multi-objective optimization algorithm is proposed to enhance system observability.
(3) Algorithm Improvement: To address the limitations of the MOPSOA and MOAOA, the NCMOGWOA incorporates nondominated sorting and a crowding distance mechanism to improve the solution accuracy. A penalty function is constructed to manage nonlinear constraints, and random starting points increase adaptability.

The remaining sections are organized as follows. The kinematic model of the UAV and the target localization system with biased bearing measurement information are detailed in the subsequent section. Then, we conduct both qualitative and quantitative analyses of the system’s observability and introduce the designed trajectory optimization method. The simulation results are provided in the penultimate section, and the conclusions are presented in the final section.

## 2. System Models

**Figure 1:**
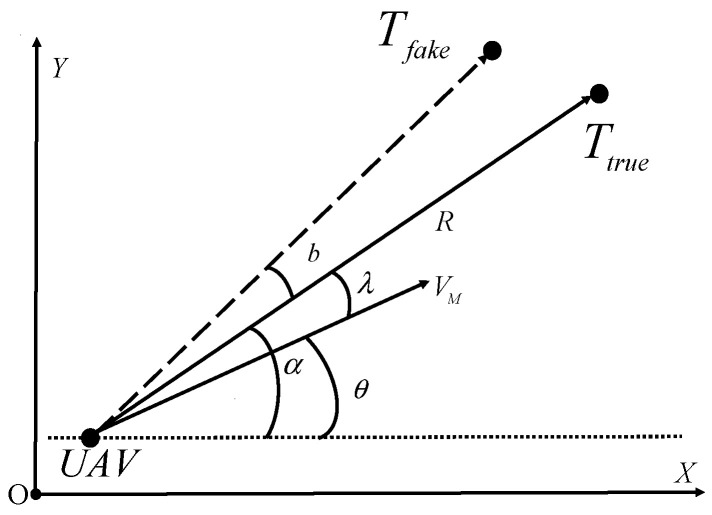
Model of the relative motion of the UAV and the target.

This paper explores a two-dimensional stationary target localization problem. As illustrated in Figure 1, the inertial reference is denoted as (X,Y). The variables with subscripts U and T indicate those of the UAV and target, respectively. The speed of the UAV is represented by V; α and R denote the bearing angle and the relative distance between the UAV and target, respectively. θ represents the flight path angle of the UAV defined in the inertial reference frame. The separation angle λ is defined as the angle between the longitudinal axis of the UAV and the line of sight of the UAV, which can be expressed as the bearing angle and flight path angle of the UAV: λ=α−θ. To ensure the uniqueness of each angle, let $\alpha ,\lambda ,\theta \in \left[-\pi ,\pi \right]$.

Assuming that the UAV moves at the same horizontal altitude as the target, the kinematics of the UAV can be formulated as follows:

(1) $$\left\{\begin{array}{l} {\dot{p}}_{U}(t)=g(\theta (t))V(t)\\ \dot{\gamma }(t)=\omega (t) \end{array}\right.$$

where $g(\theta (t))={\left[\cos\theta (t),\;\sin\theta (t)\right]}^{T}$, while $P_{U}(t)={\left[x_{U}(t),\;y_{U}(t)\right]}^{T}$ and ω(t) represent the position vector and turn rate of the UAV, respectively.

$P_{T}(t)={\left[x_{t}(t),\;y_{t}(t)\right]}^{T}$ is defined as the position vector of the target at time t in the inertial reference frame, which the UAV cannot directly obtain. The objective of target localization is to calculate $P_{T}(t)$ with the UAV’s position, speed, and bearing measurement h(t) (measured by the UAV onboard sensor). According to the literature [15], target localization with bearing information can be accurate only when affected by Gaussian noise. However, in practice, the bearing information is collected by the attitude sensor and onboard camera of the UAV, which have errors in their outputs. As shown in Figure 1, b is defined as the bias in the bearing measurement. To achieve better performance in target localization, it is necessary to estimate b to compensate for the measured bearing information; thus, the state vector X to be estimated is defined as

(2) $$X(t)={\left[x_{t}(t),y_{t}(t),b\right]}^{T}$$

The discrete-time system dynamics can subsequently be formulated as

(3) $$\left\{\begin{array}{l} X(t+1)=\Phi_{t+1,t}X(t)+w(t)\\ h(t)=\alpha (t)+b+v(t) \end{array}\right.$$

where $\Phi_{t+1,t}$ denotes the state transition matrix from t to t+1. w(t) and v(t) are the white Gaussian noise with corresponding covariances $Q_{k}$ and $L_{k}$, respectively.

$r(t)=P_{T}(t)-P_{U}\left(t\right)={\left[r_{x}(t),r_{y}(t)\right]}^{T}\in R^{2}$ is defined as the relative position vector; then, in the form of the relative position, the biased measurement function can be represented as

(4) $$h(t)=\arctan(\frac{r_{y}(t)}{r_{x}(t)})+b+v(t)$$

and the dynamic system (3) can be rewritten as

(5) $$\left\{\begin{array}{l} X(t+1)=\Phi_{t+1,t}X(t)+w(t)\\ h(t)=\arctan(\frac{r_{y}(t)}{r_{x}(t)})+b+v(t) \end{array}\right.$$

Since observability directly influences the accuracy of target estimation, this paper aims to maintain the observability of the dynamic system (5) and subsequently enhance its performance in the target localization system with biased bearing measurements.

## 3. Bio-Inspired Observability Enhancement Optimization Model

In this section, the qualitative and quantitative analyses of the system are provided, as are the observability conditions and influencing factors.

### 3.1. Qualitative Analysis of System Observability

Within the continuous-time framework, the dynamic system (5) can be replaced by

(6) $$\left\{\begin{array}{l} \dot{X}=f(X)\\ h=\arctan(\frac{r_{y}}{r_{x}})+b+v \end{array}\right.$$

**Definition** **1.** *The system (6) is observable in the time interval* [0, M] *if the initial state* $X_{0}$ *can be uniquely determined from* $h_{m}$, m∈[0, M].

Defining $H(t)={\left[h(t),\dot{h}(t),\cdots ,h^{n-1}(t)\right]}^{T}$ as the cumulative measurement vector, the arbitrary initial state X(t) and its corresponding measurement H(t) are related by

(7) H(t)=g(X(t))

According to the implicit function theorem, the unique determination of the initial state X(t) from the measurement H(t), if and only if the observability matrix, denoted by

(8) $$O(t)=\frac{\partial H(t)}{\partial X(t)}$$

is nonsingular.

**Theorem** **1.** *The dynamic system (6) is observable only if the observability* O(t) *has full rank, i.e., rank* rank(O(t))=n*, where* n *is the order of the system.*

To compute the observability matrix O(t), the Lie derivative is employed [37]. For simplicity, the state and time symbols are both ignored. The Lie derivative of h with respect to f is expressed as $L_{f}h$. The *j*-th order Lie derivative can be calculated via

(9) $$L_{f}^{j}h=\frac{\partial L_{f}^{j-1}h}{\partial X}\cdot f.$$

Consequently, the relationship between states and measurement can be represented by

(10) $$Y(t)={\left[L_{f}^{0}h,\;L_{f}^{1}h,\cdots ,\;L_{f}^{n-1}h\right]}^{T}$$

and its corresponding observability matrix can be computed by

(11) $$O(t)={\left[\frac{\partial L_{f}^{0}h}{\partial X},\;\frac{\partial L_{f}^{1}h}{\partial X},\;\cdots ,\frac{\partial L_{f}^{n-1}h}{\partial X}\right]}^{T}$$

where

(12) $$\frac{\partial L_{f}^{0}h}{\partial X}=\frac{\partial h}{\partial X},\;\frac{\partial L_{f}^{j}h}{\partial X}=\frac{\partial (\frac{\partial L_{f}^{i-1}h}{\partial X}\cdot f)}{\partial X}$$

In the dynamic system (6), the bias b remains constant, providing no additional information in the computation of the Lie derivative vector. Therefore, when computing the observability matrix, only the first two orders of O(h) are considered:

(13) $$\begin{array}{l} O=[\nabla L_{f}^{0}(h);\nabla L_{f}^{1}(h)]\\ =\left[\begin{array}{cc} -\frac{r_{y}}{r_{x}^{2}+r_{y}^{2}} & \frac{r_{x}}{r_{x}^{2}+r_{y}^{2}}\\ -\frac{-v_{y}r_{x}^{2}+2v_{x}r_{x}r_{y}+v_{y}r_{y}^{2}}{{\left(r_{x}^{2}+r_{y}^{2}\right)}^{2}} & \frac{v_{x}r_{x}^{2}+2v_{y}r_{x}r_{y}-v_{x}r_{y}^{2}}{{\left(r_{x}^{2}+r_{y}^{2}\right)}^{2}} \end{array}\right] \end{array}$$

For simplicity, let $R=\left\|r\right\|\;,\;V=\left\|v\right\|$. Hence, in polar coordinates, we have

(14) $$\left\{\begin{array}{l} r_{x}=R\cos\alpha \\ r_{y}=R\sin\alpha \end{array}\right.\;\;\;,\;\;\;\left\{\begin{array}{l} v_{x}=V\cos\theta \\ v_{y}=V\sin\theta \end{array}\right.$$

By substituting (14) into (13), the observability matrix can be simplified as

(15) $$O=\left[\begin{array}{cc} -\frac{\sin\left(a\right)}{R} & \frac{\cos\left(a\right)}{R}\\ \frac{V\sin\left(2a-\theta \right)}{R^{2}} & -\frac{V\cos\left(2a-\theta \right)}{R^{2}} \end{array}\right]$$

and its corresponding determinant can be computed as

(16) $$\det(O)=-\frac{RV\sin\left(a-\theta \right)}{R^{4}}$$

According to Theorem 1, the system (6) is observable only when the observability matrix O has full rank, which is equivalent to the fact that the determinant of O is nonzero, i.e.,

(17) $$\det(O)\neq 0\;\;\Rightarrow \;\;\left\{\begin{array}{l} \lambda =\alpha -\theta \neq 0\\ V\neq 0 \end{array}\right.$$

When V=0, the UAV remains stationary, and the bearing measurement cannot be updated; when α−θ=0, $r(t+1)=k_{1}\;r(t)\;,\;k_{1}\in {\mathbb{R}}^{+}$. Regardless of the angle measurement, distinguishing the next step relative position vector from the current step relative position vector is impossible. Hence, to ensure system observability, the separation angle λ and the relative speed between the UAV and the target must remain nonzero.

### 3.2. Bio-Inspired Unobservable Area Avoidance Based on the CBF Method

The observability matrix O is determined by the bearing angle α, the relative distance R, the speed V, and the flight path angle θ of the UAV. When the UAV flight parameters are constant, the observability matrix O can be uniquely determined by the relative positional relationship between the UAV and the target.

**Definition** **2.** *The observability of the dynamic system can be determined by the geometric relationship between the UAV and the target.*

To guarantee the observability of the dynamic system, it is crucial to modify the geometric relationship between the UAV and the target. In this work, inspired by phototropism and plants’ dark avoidance behavior, the CBF was employed to categorize the observable and unobservable areas. Additionally, restrictions were imposed to ensure UAV movement within the observable areas and maintain the system’s observability.

**Figure 2:**
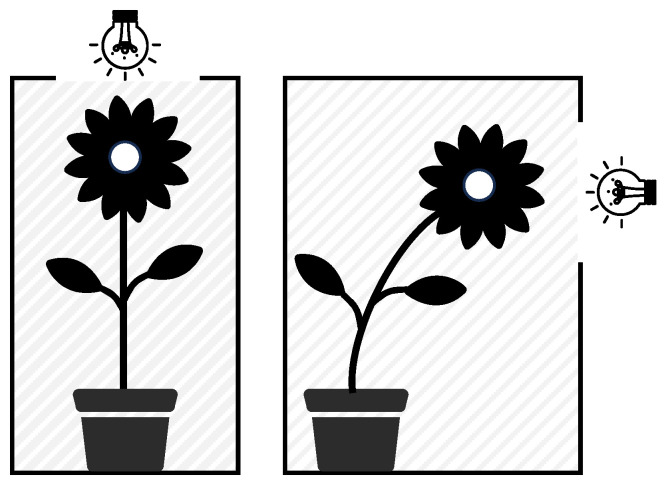
Phototropism in plants, where the bulb represents the light source.

Phototropism refers to the phenomenon in which plants bend toward light when they are exposed to it [38,39]. It is considered to be a mechanism by which plants adapt to low-light environments, as illustrated in Figure 2.

**Definition** **3.** *For a smooth function* h:D→R*, define* C *as a superlevel set of* h*, its boundary as* ∂C*, and its interior as* int(C)*:*

(18) $$\begin{array}{r} C=\left\{x\in D\subset R^{n}:B(x)\geq 0\right\}\\ \partial C=\left\{x\in D\subset R^{n}:B(x)=0\right\}\\ \mathrm{int}(C)=\left\{x\in D\subset R^{n}:B(x)>0\right\} \end{array}$$

*Considering a general nonlinear system*

(19) $$\dot{x}=f(x)+g(x)\;u$$

*if there exists a constant* δ>0* such that for all* x∈D*, satisfying*

(20) $$\begin{array}{l} \sup\left[L_{f}h(x)+L_{g}h(x)\;u+\delta h(x)\right]\geq 0\\ i.e.,\sup\left[\dot{h}(x)\;u+\delta h(x)\right]\geq 0 \end{array}$$

*then,* h *is a control barrier function of (19).*

The control barrier function is frequently employed for addressing safety analysis and control issues in nonlinear systems [40,41]. In this work, the control barrier function was employed to delineate observable and unobservable areas and restrict UAV motion, thereby ensuring system observability.

From (1), the kinematics of the UAV can be rewritten as

(21) $$\begin{array}{l} {\dot{X}}_{U}=f(X_{U})+g(X_{U})u\\ =\left[\begin{array}{c} \sin(\theta )\\ \cos(\theta )\\ 0 \end{array}\right]V+\left[\begin{array}{l} 0\\ 0\\ 1 \end{array}\right]w \end{array}$$

where $X_{U}={\left[x_{u},\;y_{u},\;\theta \right]}^{T}$.

By utilizing the system observability conditions provided in (16) and (17), the control barrier function can be formulated as

(22) $$B(x)={\left(RV\sin(\alpha -\theta )\right)}^{2}$$

The CBF observability constraints can subsequently be established through the following inequality:

(23) $$\begin{array}{c} \dot{B}(x)+\delta \;B(x)\geq 0\\ i.e.,\;L_{f}B+L_{g}B\;u+\delta \;B(x)\geq 0 \end{array}$$

where δ∈(0, 1) represents the attenuation rate of B(x). $L_{f}B$ and $L_{g}B$ denote the Lie derivatives along the vector fields f and g, respectively:

(24) $$L_{f}B=\nabla B(x)f,\;L_{g}B=\nabla B(x)g$$

(25) $$\nabla B(x)=\left[\begin{array}{c} \;\;2rV^{2}\sin(\alpha -\theta )\sin\theta \\ -2rV^{2}\sin(\alpha -\theta )\cos\theta \\ 0 \end{array}\right]$$

Substituting (24) and (25) into (23) yields the observability constraints of the system:

(26) $$R^{2}V^{2}\sin(2\theta -2\alpha )w+\delta {\left(RV\sin(\alpha -\theta )\right)}^{2}\geq 0$$

### 3.3. Influence of δ on System Observability

By integrating (23) from t to t+Δt, we have

(27) $$B(x(t+\Delta t))\geq B(x(t))-\int_{t}^{t+\Delta t}\delta B(x(\tau ))d\tau$$

Subsequently, the superlevel set $S_{\Delta t}$ that satisfies the CBF observability constraint at x(t+Δt) can be defined as

(28) $$S_{\Delta t}=\left\{x\in {\mathbb{R}}^{n}:B(x(t+\Delta t))\geq B(x(t))-\int_{t}^{t+\Delta t}\delta B(x(\tau ))d\tau \right\}$$

**Figure 3:**
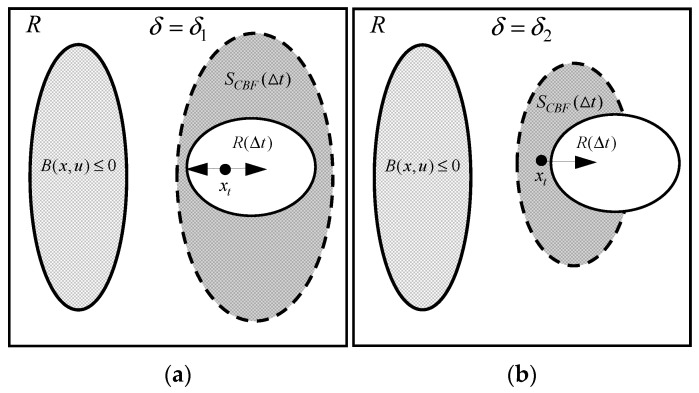
Effect of δ ( $0<\delta_{2}<\delta_{1}<1$) on system observability: (**a**) $\delta =\delta_{1}$; (**b**) $\delta =\delta_{2}$. The gray oval with a solid outline denotes the unobservable area. The gray oval with a dashed outline and the white oval with a solid outline represent $S_{\Delta t}$ and $R_{\Delta t}$, respectively. The black dot represents x(t).

Let $R_{\Delta t}$ denote the set of states reachable by the system after Δt when the constraints are satisfied. Hence, a solution exists at x(t) if the UAV’s motion at x(t) fulfills both the control input constraints and the observability CBF constraints, i.e., $R_{\Delta t}\cap S_{\Delta t}\neq 0$. The impact of δ on system observability is illustrated through the geometric relationship between $R_{\Delta t}$ and $S_{\Delta t}$, as shown in Figure 3.

When $\delta =\delta_{1}$, as illustrated in Figure 3a, and $S_{\Delta t}\cap R_{\Delta t}=R_{\Delta t}$, the UAV has the flexibility to either approach or move away from the unobservable area, while ensuring the system’s observability at x(t+Δt). However, the possibility of the UAV moving closer to the unobservable area increases the risk of the system becoming unobservable.

When $\delta =\delta_{2}$, $S_{\Delta t}\cap R_{\Delta t}\neq 0$, as shown in Figure 3b, the UAV is restricted to moving far from the unobservable area, ensuring observability. However, as the coverage area of $S_{\Delta t}\cap R_{\Delta t}$ decreases, the feasible area for UAV motion diminishes.

From Figure 3a,b, it is evident that with a certain value of Δt, as δ decreases, as indicated by (28), the area of $S_{\Delta t}$ diminishes. This compression reduces the area covered by $S_{\Delta t}\cap R_{\Delta t}$, thereby decreasing the likelihood of the UAV moving closer to the unobservable area and ensuring system observability.

### 3.4. Quantitative Analysis of System Observability

In addition to maintaining the system’s observability, we aim to enhance it to improve the state estimation performance. To analyze system observability quantitatively, $C^{*}$ was defined as the observability metric:

(29) $$C^{*}=\frac{\xi_{\max}(O)}{\xi_{\min}(O)}$$

where $C^{*}$ represents the condition number of the observability matrix O. $\xi_{\min}(O)$ and $\xi_{\max}(O)$ denote the minimum and maximum singular values, respectively.

**Definition** **4.** *A system is considered to have weak observability if the condition number of the observability matrix is extremely large or infinite.*

**Definition** **5.** *A system is considered to have strong observability if the condition number of the observability matrix is close to one.*

Substituting (15) into (29), we have

(30) $$C^{*}=\sqrt{\frac{V^{2}+R^{2}+\sqrt{{\left(V^{2}+R^{2}\right)}^{2}-4V^{2}R^{2}\sin{\left(a-\theta \right)}^{2}}}{V^{2}+R^{2}-\sqrt{{\left(V^{2}+R^{2}\right)}^{2}-4V^{2}R^{2}\sin{\left(a-\theta \right)}^{2}}}}$$

(30) indicates that $C^{*}\geq 1$, $C^{*}=1$ if and only if the following equation is satisfied:

(31) $${\left(V^{2}+R^{2}\right)}^{2}=4V^{2}R^{2}\sin{\left(a-\theta \right)}^{2}\Rightarrow \;\;1+\frac{R^{2}}{V^{2}}=2\frac{R}{V}\left|\sin\left(a-\theta \right)\right|$$

Typically, the relative distance between the UAV and target significantly exceeds the speed of the UAV, i.e., R≫V. Consequently, $1+R^{2}/V^{2}$ is generally much larger than $2R/V\cdot \left|\sin\left(a-\theta \right)\right|$, and Equation (31) can be satisfied only when R/V=1 and α−θ=±π/2.

**Figure 4:**
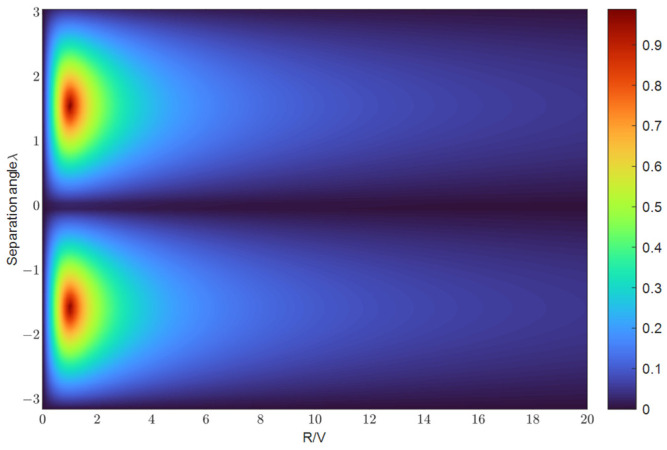
Heatmap of the inverse of condition number. The horizontal coordinate represents the ratio of the relative distance between the UAV and the target to the speed of the UAV. The vertical coordinate represents the angle of separation.

By defining $c=1/C^{*}$, a heatmap was generated to visualize the distribution of c. As demonstrated in Figure 4, when R/V is closer to 1 and λ is closer to ±π/2, $C^{*}$ is closer to 1, indicating improved system observability. Consequently, to enhance the observability, the UAV must approach the target closely, while also adjusting the flight path angle θ to make the separation angle λ approach ±π/2.

### 3.5. Trajectory Optimization Model

Based on the observability analysis above, a multi-objective optimization model was formulated to enhance the observability of the dynamic system while ensuring it. The object functions were defined as $f_{1}=\left\|R/V-1\right\|$ and $\;f_{2}=\left\|abs(\lambda )-\pi /2\right\|$, respectively. Typically, the distance from the UAV to target R is significantly greater than the speed of the UAV; the proximity of R/V and 1 is comparable to the proximity of R and 1. Hence, considering a minimum safe distance $R_{safe}$ between the UAV and the target, the first objective function can be redefined as $f_{1}=\left\|R-R_{safe}\right\|$.

(32) $$\begin{array}{l} \;\;\;\;\min\;\;\;\;\;\;\;\;\;\;\;f_{1}=\left\|R-R_{safe}\right\|\\ \;\;\;\;\min\;\;\;\;\;\;\;\;\;\;\;f_{2}=\left\|abs(\lambda )-\pi /2\right\|\\ \mathrm{subject}\;\mathrm{to}\left\{\begin{array}{l} r^{2}V^{2}\sin(2\theta -2\alpha )\omega +\delta {\left(rV\sin(\alpha -\theta )\right)}^{2}\geq 0\\ \;\;\;\;\;\;\;\;\;\;v_{\min}\leq v\leq v_{\max}\\ \;\;\;\;\;\;\;\;\;\;\omega_{\min}\leq \omega \leq \omega_{\max}\\ \;\;\;\;\;\;\;\;\;\;\;\;\;\;\;\;\;R_{safe}\leq R \end{array}\right. \end{array}$$

where $v_{\min}$ and $v_{\max}$ denote the minimum and maximum UAV speeds, respectively; $\omega_{\min}$ and $\omega_{\max}$ denote the minimum and maximum UAV turn rates, respectively.

Although the optimization problem in (32) can be solved via traditional linear optimization techniques, heuristic methods provide greater flexibility and adaptability, especially when searching for local optimal solutions. To improve the convergence speed, we utilized the NCMOGWOA algorithm in this study. As will be shown in Section 5, our simulations highlight the superior convergence speed of the NCMOGWOA algorithm compared with other methods, demonstrating its effectiveness for the given optimization problem.

## 4. Nonlinear Constrained Multi-Objective Gray Wolf Optimization Algorithm (NC-MOGWOA)

The optimization model presented in the last section constitutes a multi-objective, nonlinear programming problem. As the number of candidates in the state space increases, the potential combinations available for selection rise exponentially, posing a challenge for solutions using conventional methods [42,43].

The Gray Wolf Optimization Algorithm (GWOA) is a bio-inspired algorithm that simulates the predatory actions of gray wolf populations in nature [44]. It efficiently tracks the optimal solution’s iterative direction and finds the optimal solution, enabling quick discovery. In this paper, a Nonlinear Constrained Multi-Objective Gray Wolf Optimization Algorithm (NCMOGWOA) is employed to address the presented multi-objective, nonlinear programming problem.

### 4.1. Gray Wolf Optimization Algorithm (GWOA)

The GWOA is a meta-heuristic algorithm inspired by the predatory behavior observed in gray wolf populations. It combines the hierarchy and distribution patterns observed within these populations to simulate the hunting and encircling process of gray wolves when they pursure their prey. This process includes four steps: establishing social hierarchies, searching for prey, encircling prey, and attacking prey.

The wolves are classified into 4 distinct classes: $\partial_{1}$, $\partial_{2}$, $\partial_{3}$, and $\partial_{4}$, and each class has unique responsibilities within the pack. Wolf $\partial_{1}$ possesses managerial skills and oversees decisions regarding food acquisition and location; wolf $\partial_{2}$ aids in decision making and serves as a communicator; wolf $\partial_{3}$ follows the directives of wolf $\partial_{1}$ and wolf $\partial_{2}$, undertaking tasks such as scouting and guarding; and wolf $\partial_{4}$ complies with the pack’s hierarchy, maintaining social equilibrium. The GWO model can be expressed as

(33) $$D=\left|C\cdot X\right|$$

(34) $$X(t+1)=X_{p}(t)-A\cdot D$$

(35) $$A=2a\cdot r_{1}-a$$

(36) $$C=2\cdot r_{2}$$

(37) $$\left\{\begin{array}{l} D_{\partial 1}=\left[C_{1}\cdot X_{\partial 1}-X\right]\\ D_{\partial 2}=\left[C_{2}\cdot X_{\partial 2}-X\right]\\ D_{\partial 3}=\left[C_{3}\cdot X_{\partial 3}-X\right] \end{array}\right.$$

(38) $$\left\{\begin{array}{l} X_{1}=X_{\partial 1}-A_{1}\cdot D_{\partial 1}\\ X_{2}=X_{\partial 2}-A_{2}\cdot D_{\partial 2}\\ X_{3}=X_{\partial 3}-A_{3}\cdot D_{\partial 3} \end{array}\right.$$

(39) $$X(t+1)=(X_{1}+X_{2}+X_{3})/3$$

where D represents the distance between the wolf and the prey, while $X_{p}$ and X denote the positions of the prey and the wolf, respectively. Both A and C are coefficient vectors. The parameter a denotes the convergence factor, which linearly decreases with each iteration, and $r_{1}$ and $r_{2}$ are randomly selected values within the range [0, 1].

In the hunting process, wolves $\partial_{1}$, $\partial_{2}$, and $\partial_{3}$ initially make a random estimation of the prey’s location, since it is unknown. They then guide the other wolves to assess and update the estimated location iteratively until an optimal solution is achieved.

The coefficient vector A, with a range of [−2a, 2a], influences the wolf’s decision making regarding its current position relative to that of the prey. More precisely, when $\left|A\right|>1$, the algorithm exhibits a robust search capability, causing the wolf to move farther away from the prey. Conversely, when $\left|A\right|\leq 1$, the algorithm shows a strong developmental ability, prompting the wolf to move closer to the prey.

### 4.2. Multi-Objective Gray Wolf Optimization Algorithm

To adapt the GWOA to multi-objective problems, two enhancements were introduced to the algorithm.

#### 4.2.1. External Stock Archive

An external population archive was introduced to store nondominated solutions. At each iteration, the algorithm generates a new position for the gray wolf. The new gray wolves are compared to the original gray wolves stored in the archive when their eligibility for the archive is assessed. If the new gray wolf is dominated by all the original wolves in the archive, it cannot join the pack. Conversely, if the new gray wolf dominates one or more gray wolves, it joins the pack, displacing any dominated wolf. If neither dominates the other, the new gray wolf can join only if the archive has not reached its maximum capacity.

#### 4.2.2. Decision-Making Wolf Selection

In the literature [45], a roulette method was employed to choose the decision-making wolf from the archive. This method involves identifying the least crowded grid in the archive and randomly selecting three solutions corresponding to wolves $\partial_{1}$, $\partial_{2}$, and $\partial_{3}$, without any perceived superiority or inferiority. If the number of segments is insufficient, the selection is deferred to the grid with the second lowest crowdedness.

### 4.3. Nonlinear Constraint Penalty Function

Multi-objective optimization problems frequently involve nonlinear inequality and equation constraints, rendering them challenging to solve. However, the Multi-Objective Gray Wolf Optimization Algorithm (MOGWOA) does not account for these nonlinear constraints, potentially causing it to exceed the allowable boundaries during wolf location updates [45]. In this paper, based on the MOGWOA, we exclusively consider nonlinear inequality and equation constraints. A general model for multi-objective programming problems can be expressed as

(40) $$\begin{array}{l} \min\;\;f_{1}(x),\;f_{2}(x),\cdots f_{n}(x)\\ s.t\left\{\begin{array}{l} \;\;\;\;x_{i}^{L}\leq x_{i}\leq x_{i}^{U}\\ g_{m}(x)=0,\;m=1,\;2,\;\cdots ,\;j\\ \;f_{q}(x)\leq 0,\;q=1,\;2,\;\cdots ,\;k \end{array}\right. \end{array}$$

where $g_{m}(x)$ and $f_{m}(x)$ represent the nonlinear equational and inequality constraints, respectively. The constraint penalty function ρ(x) can be defined as follows:

(41) $$\rho (x_{i})=\sum_{m=1}^{j}p_{g}\cdot g_{m}(x_{i})+\sum_{q=1}^{k}p_{f}\cdot f_{q}(x_{i})\cdot H(x_{i})$$

where $p_{f}$ and $p_{f}$ are constants, and where H(x) is a judgment function of inequality constraint f(x):

(42) $$H(x)=\left\{\begin{array}{l} 0,\;\;\;\;\;\;\;f(x)\leq 0\\ 1,\;\;\;\;\;\;\;f(x)>0 \end{array}\right.$$

With the introduction of the constraint penalty function, the nonlinear constraints can be converted into the objective function, thereby simplifying the problem-solving process. The multi-objective programming problem can be reformulated as

(43) $$\begin{array}{l} \min\;\;f_{1}(x)+\rho (x),\;\;f_{2}(x)+\rho (x),\cdots f_{n}(x)+\rho (x)\\ s.t\left\{\begin{array}{l} \;\;\;\;x_{i}^{L}\leq x_{i}\leq x_{i}^{U}\\ g_{m}(x)=0,\;m=1,\;2,\;\cdots ,\;j\\ \;f_{q}(x)\leq 0,\;q=1,\;2,\;\cdots ,\;k \end{array}\right. \end{array}$$

As represented in (43), if the updated position of the wolves breaches the nonlinear constraints, the values of each objective function significantly increase, rendering the wolves’ location nonoptimal. In other words, the optimal solution in each iteration must satisfy all the constraints.

Algorithm 1 demonstrates the process of the proposed NCMOGWOA. In line 1, the process begins with the random initialization of the wolf population through the following equation:

(44) $$X_{i}=X_{\min}+rand(0,1)\cdot (X_{\max}-X_{\min})$$

where i=1,2,⋯, N, rand(0,1) represents a uniformly distributed random number in the range [0, 1]; $X_{\min}$ and $X_{\max}$ denote the lower and upper bounds of the dimension, respectively.
**Algorithm 1:** Nonlinear Constrained Multi-Objective Gray Wolf Optimization Algorithm (NCMOGWOA)1:***begin***2: *Selected the gray wolf population Xi randomly selected within the feasible region (i = 1, 2, …, n)*3: *Initialize a, A, and C using Equations (35) and (36).*4: *Calculate the objective values for each search agent using Equation (40)*5: *The initial archive Ar_0_ ← The nondominated solutions*6:*X∂1, X∂2, X∂3 ← The initial archive A_0_ (select the initial three wolves with the lowest objective*7:*function values)*8: ***for***
*t = 1,2, …, Max number of iterations*9:  *Update the positions of each current search agent using Equations (37), (38) and (39)*10:  *Update a, A, and C using Equations (35) and (36)*11:  *Calculate [f1(x), f2(x), …fn(x)] of all search agents using Equation (40)*12:  *Calculate ρ(x) for all search agents using Equation*
13:  *[f1(x), f2(x), …fn(x)] ← [f1(x) + ρ(x), f2(x) + ρ(x), …fn(x) + ρ(x)]*14:  *The archive A ← The nondominated solutions*15:  ***If***
*the archive is full*16:   *Omit one of the current archive members*
17:*The archive Ar ← The archive Ar + The new solution*18:***end if***19:***If*** *solutions are outside the hypercubes*20:*Update the grids to cover the new solutions*21:***end if***22:*X∂1, X∂2, X∂3 ← The archive Ar*23:***end for***24:***end***25:***return*** *archive Ar*

The proposed algorithm uses the archive for storing the nondominated solution. For each iteration in the loop, after generating a new wolf population, the penalty function is calculated along with all the objective functions (lines 11, 12). The objective function is updated by adding the penalty function to the original objective function values (line 13).

All the current new gray wolves are compared with the original gray wolves stored in the archive in terms of the updated objective functions. If the new gray wolf is dominated by all the original wolves in the archive, it cannot join the pack. Conversely, if the new gray wolf dominates one or more gray wolves, it joins the pack, displacing any dominated wolf (lines 14–18).

After the search process, wolves $\partial_{1}$, $\partial_{2}$, and $\partial_{3}$ are replaced by the three best wolves in the current archive, respectively (line 22). When the algorithm finishes, the updated archive is stored as the output, which stores all the nondominated solutions.

### 4.4. Selection of the Optimal Solution

As the NCMOGWOA yields a Pareto optimal solution, it becomes challenging to simultaneously obtain optimal solutions for both objectives $f_{1}$ and $f_{2}$, thus complicating the selection of optimal UAV inputs.

**Definition** **6.** *Given a cost-based, multi-objective optimization problem with a feasible solution set* Ω*:* min f(X)*,* X∈Ω*, if* $X^{*}\in \Omega$ *satisfies.*

(45) $$\underset{i\in I}{\Lambda }(f_{i}(X)\geq f_{i}(X^{*}))$$

Consequently, $X^{*}$ is denoted as the optimal solution of min f(X).

**Definition** **7.** *Given a cost-based, multi-objective optimization problem with a feasible solution set* Ω*:* min f(X)*,* X∈Ω*, if there exists* $X^{*}\in \Omega$ *and no other* ${\bar{X}}^{*}\in \Omega$*, then (46) holds.*

(46) $$f_{j}(X^{*})\geq f_{j}({\bar{X}}^{*}),\;(j=1,2,\ldots ,r)$$

and at least one of (46) is a strict inequality; then $X^{*}$ is denoted as the Pareto optimal solution of min f(X).

**Figure 5:**
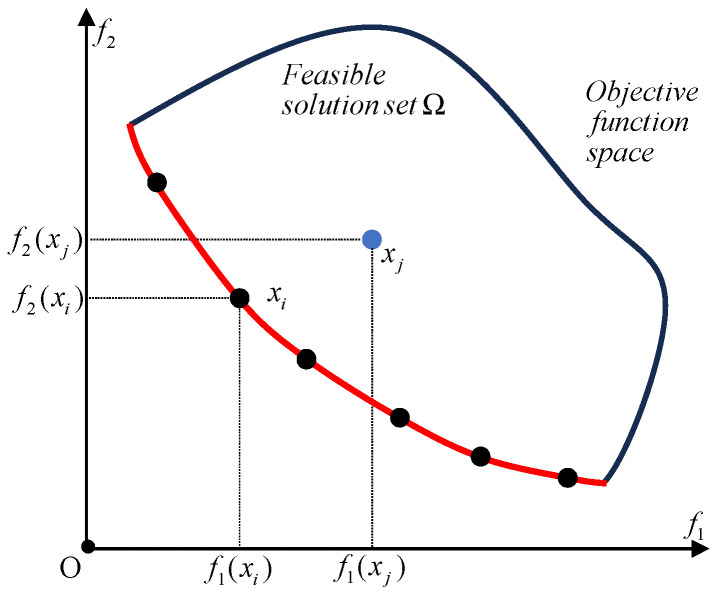
The Pareto solution of the two objective optimization problems. The black dots denote the Pareto solution while the blue dots do not. The red line denotes the Pareto front.

The set containing all the Pareto optimal solutions of min f(X) is referred to as the Pareto optimal solution set of min f(X). The graphical representation of the Pareto optimal solution set in the space of the objective function is defined as the Pareto front. These concepts are demonstrated in Figure 5.

Figure 5 illustrates the Pareto solution of a two-objective optimization problem. Each black dot within the feasible solution set Ω represents a Pareto solution, collectively forming the Pareto solution set. The red line indicates this front, highlighting solutions where neither objective $f_{1}(x)$ nor $f_{2}(x)$ can be improved without sacrificing the other. Thus, $x_{j}$ is not a Pareto solution.

To provide a comprehensive evaluation, a synthesized evaluation methodology that combines Technique for Order Preference by Similarity to Ideal Solution (TOPSIS) and the Criteria Importance Through Intercriteria Correlation (CRITIC) method (TCM) was proposed. Applying the TCM to the Pareto front solution set obtained by the NCMOGWOA enables it to determine the optimal solution to the problem.

In this paper, the CRITIC method is employed to assign weights to each objective function in every iteration. CRITIC is usually used to weight indicators. It considers the differences and similarities among evaluation indicators, assigning smaller weights to indicators with high horizontal similarity and larger weights to those with significant vertical differences [46].

With the weights generated by the CRITIC method, the TOPSIS method was employed to synthesize the assessments of various objective function values. TOPSIS chooses the optimal solution by establishing the positive and negative ideal solutions of the evaluation problem and the optimal and worst solutions for each index. It ranks the solutions based on their relative closeness to the ideal solution, considering their proximity to the positive and negative ideal solutions [47].

**Figure 6:**
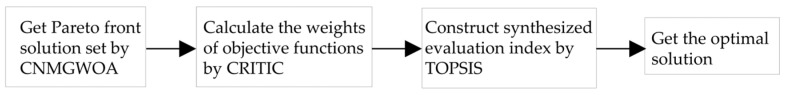
Process of the TOPSIS and CRITIC methods (TCM).

The TCM process is illustrated in Figure 6. Once the Pareto front solution set was obtained by the NCMOGWOA in each iteration, the weight of each objective function could be computed using the CRITIC method. Based on these weights, the synthesized evaluation index was subsequently constructed using the TOPSIS method, and the final scores were derived for all Pareto solutions. We selected the optimal solution and the corresponding optimized variables from these scores.

Upon examining the distribution of c in Figure 4, it is evident that during the initial phase, the impact of R on the condition number is almost negligible due to its large magnitude. Consequently, it is imperative to prioritize the optimization of $f_{1}$ to ensure a decrease in the value of $C^{*}$ to an appropriate level.

However, solely concentrating on reducing the distance R can lead the UAV to violate the CBF constraint. Therefore, the optimization objectives of this problem are divided into two stages:

(47) $$Solution_{opt}=\left\{\begin{array}{l} Solution_{R}\;,\;R>R_{switch}\\ {Solution}_{TCM},\;R\leq R_{switch} \end{array}\right.$$

where $Solution_{opt}$ represents the optimal solution, $Solution_{R}$ represents the Pareto optimal solution with the smallest value in $f_{1}$, ${Solution}_{TCM}$ indicates the optimal solution selected by the TCM, and $R_{switch}$ is a constant.

**Figure 7:**
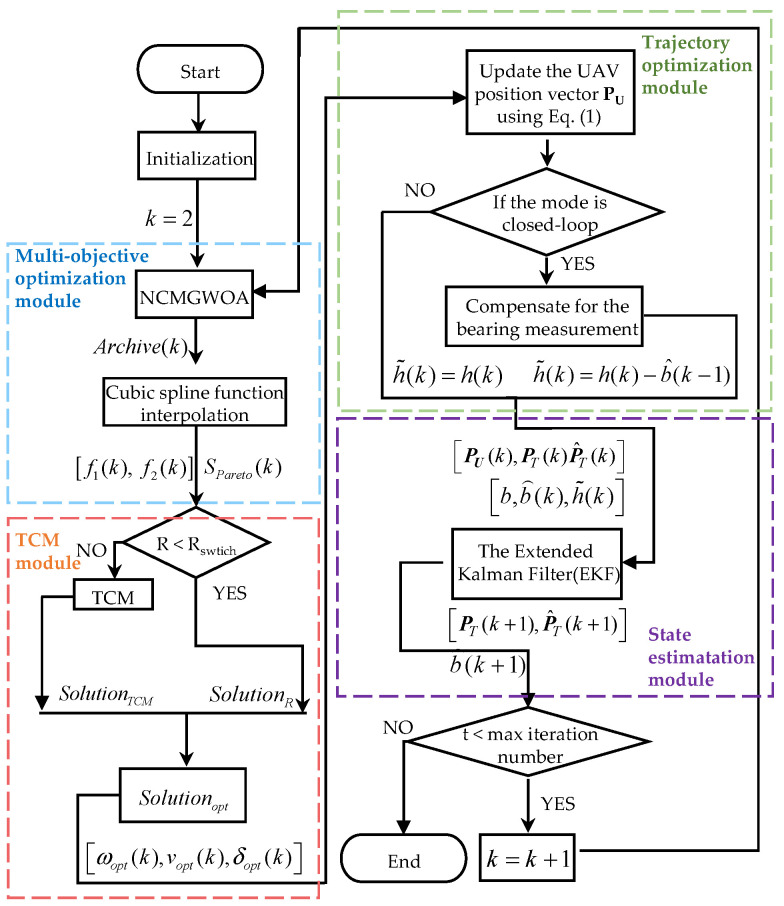
Flowchart of the algorithm for biased bearing information-only target localization and UAV trajectory optimization based on observability enhancement.

Figure 7 demonstrates an algorithm for biased bearing information-only target localization. The aim was to optimize the UAV trajectory to improve the localization performance. First, by employing the NCMOGWOA, the optimal Pareto set $S_{pareto}$ was obtained, as was its corresponding objective function value vector $f_{1},\;f_{2}$. Afterward, utilizing the optimal Pareto set $S_{pareto}$ with the TCM, we selected the optimal solution according to (47), which was then used to update the position of the UAV. Furthermore, employing the Extended Kalman Filter (EKF) facilitated obtaining both ${\hat{P}}_{T}$ and $\hat{b}$, where ${\hat{P}}_{T}$ and $\hat{b}$ denote the estimation of the target’s position and bias in the bearing measurement h(t), respectively. In closed-loop mode, $\hat{b}$ was employed to compensate for the bearing measurement h(t), enhancing its accuracy.

## 5. Experiments and Results

In this section, the bearing-only stationary target localization problem is solved by the proposed UAV trajectory optimization algorithm based on observability enhancement. We analyze the differences between open-loop and closed-loop modes within the algorithm and explore the effects of varying δ and initial flight path angles on trajectory optimization. Finally, we evaluate the proposed NCMOGWOA’s localization accuracy and convergence by comparing it with the MOPSOA [34], MOAOA [35], and SQP methods [36]. The comparison is conducted through state estimation and the GD and IGD metrics. The simulation platform for each experiment in this study is the Windows 11 AMD Ryzen 5 5600H chip system, and the MATLAB version is R2021b.

### 5.1. Trajectory Optimization Results for Target Localization

In the case of a stationary target, the state transition matrix $\Phi_{t+1,t}$ can be represented by an identity matrix. The relevant parameters are summarized in Table 1.

In this study, the target position and bias bearing measurement bias were estimated using the Extended Kalman Filter (EKF). The initial value for state estimation was set as follows:

(48) $$X_{0}={\left[470\;m\;\;\;\;470\;m\;\;\;\;3^{{}^{\circ}}\right]}^{T}$$

The covariance ***Q*** and initial values of the error covariance matrix were set to

(49) $$Q=\mathrm{diag}({\left(0.1\;m\right)}^{2}\;\;\;{\left(0.1\;m\right)}^{2}\;\;\;{\left(0.001^{{}^{\circ}}\right)}^{2})$$

(50) $$P_{0}=\mathrm{diag}({\left(25\;m\right)}^{2}\;\;\;{\left(25\;m\right)}^{2}\;\;\;{\left(0.1^{{}^{\circ}}\right)}^{2})$$

The target localization system, which relies on biased bearing measurements, operates in closed-loop mode. In this mode, the estimation of the bearing measurement bias b at each moment compensates for the subsequent bearing measurement, reducing its impact. Accurate estimation of bias b improves the precision of bearing measurements. In closed-loop mode, based on (4), the measurement function at each time instant t is described as follows:

(51) $$h_{close}(t)=h(t)-\hat{b}(t-1)$$

where $\hat{b}(t-1)$ denotes the estimation of bias b at time instant t−1.

**Figure 8:**
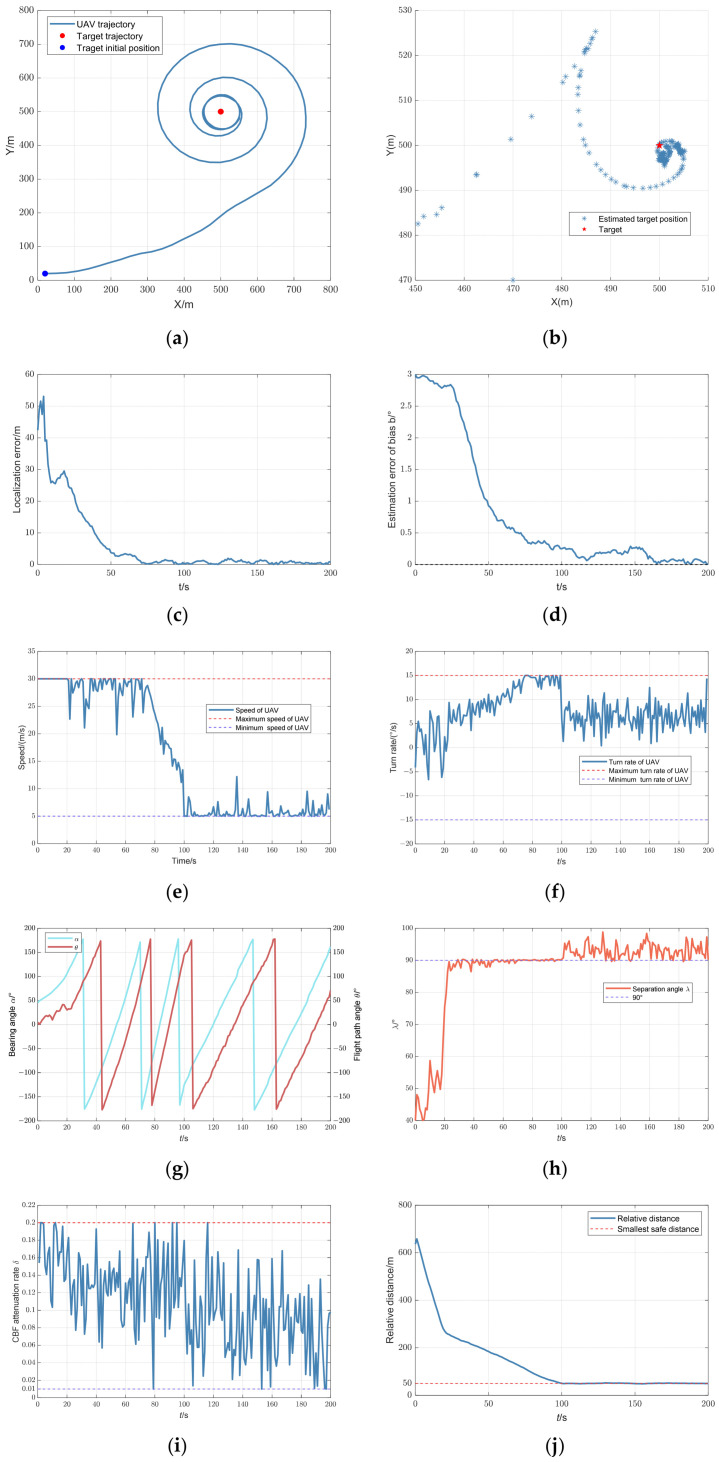

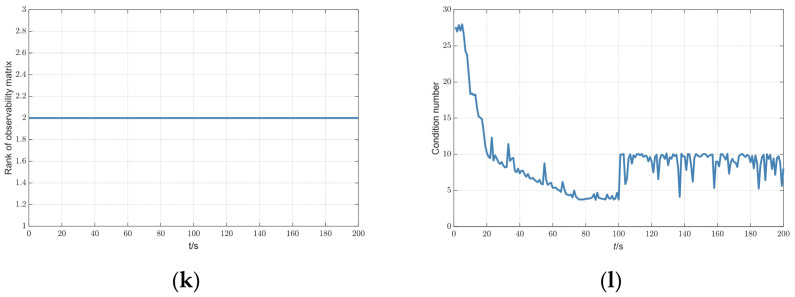
UAV trajectory optimization results for a stationary target: (**a**) trajectory of UAV; (**b**) estimated position of target; (**c**) localization error; (**d**) estimation error of bias b; (**e**) speed of UAV; (**f**) turn rate of UAV; (**g**) bearing and flight path angle; (**h**) separation angle; (**i**) attenuation rate of CBF; (**j**) relative distance; (**k**) rank of observability matrix; and (**l**) condition number.

The trajectory optimization results are illustrated in Figure 8. In Figure 8a, the UAV trajectory forms a decreasing radius circle around the stationary target. When $R>R_{switch}$, the UAV approaches the target, increasing the separation angle λ. As R rapidly decreases to $R_{switch}$, there is a sharp decline in the condition number, as shown in Figure 8l. At $R_{switch}$, ${Solution}_{TCM}$ is selected as the optimal solution. To maintain the separation angle λ near π/2, the UAV continues a circular path with a diminishing radius, enhancing system observability. When R reaches the safe distance $R_{safe}$, the UAV slows down to maintain this distance, orbiting the target at $R_{safe}$.

Figure 8c,d,k,l demonstrates that the observability matrix remained full rank throughout. The localization and bearing bias estimation errors converged to zero as the condition number decreased. Moreover, the estimated position of the target eventually converged to its real position, as shown in Figure 8b. As R stabilizes, the speed of the UAV must decrease, causing a slight increase in the condition number. However, these fluctuations were minimal, and had a negligible effect on estimation accuracy. It is evident that the proposed trajectory algorithm had a significant effect on observability enhancement, resulting in accurate localization of the target and calibration of the bearing measurement.

### 5.2. Comparison between the Open-Loop and Closed-Loop Modes

This section analyzes the performance differences in trajectory optimization between the open-loop and closed-loop modes, focusing on whether the estimation of bias *b* is used to compensate for the target bearing measurement. The simulation parameters for both modes remain consistent with the values provided in Table 1.

**Figure 9:**
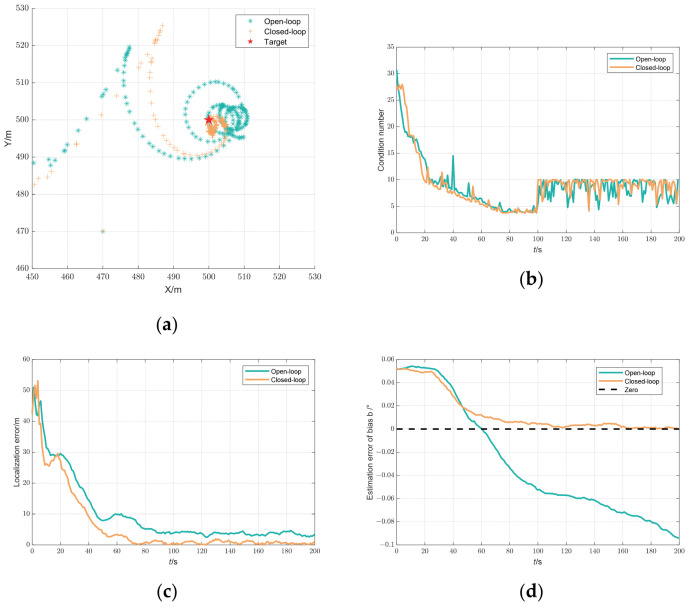
UAV trajectory optimization results between the open-loop and closed-loop mode: (**a**) estimated location of the target; (**b**) condition number; (**c**) localization error; and (**d**) estimation error of bias b.

For simplicity, we denoted the open-loop mode as $Mode_{open}$ and the closed-loop mode as $Mode_{closed}$. Figure 9 shows that the trends in the condition number were similar for both modes. However, a significant difference exists in the estimation of bias b: the error can converge to nearly zero at $Mode_{closed}$ but not at $Mode_{open}$. Figure 9a,c and Table 2 demonstrate the superior performance of $Mode_{closed}$ in target localization, where the estimated target location converged to the actual position, unlike in $Mode_{open}$. Moreover, except for the initial period, the localization error in $Mode_{closed}$ remained lower than $Mode_{open}$ and could converge to zero.

### 5.3. Effect of δ on Trajectory Optimization

**Figure 10:**
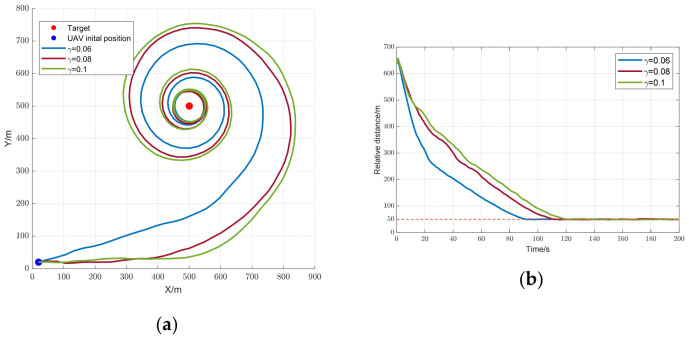

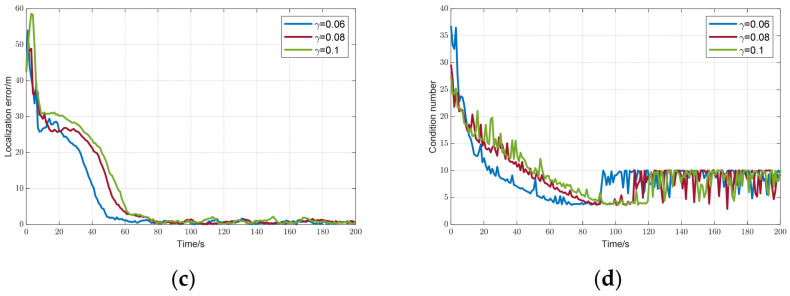
Trajectory optimization results for different δ values: (**a**) trajectory of UAV; (**b**) relative distance; (**c**) localization error; and (**d**) condition number.

To explore the effect of δ on trajectory optimization, the attenuation rates δ of the CBF were divided into three groups, namely, $\delta_{1}=0.06,\;\;\delta_{2}=0.08,\;\;\delta_{3}=0.1$, instead of being optimized as a parameter by the NCMOGWOA. To exclude other factors, the optimal solution was chosen by the TCM throughout the process. The simulation results are presented in Figure 10.

Figure 10a,b illustrates that as δ decreased, the constraints imposed by the CBF on the UAV were reduced. A smaller δ allowed the UAV to approach the target more rapidly, leading to a quicker reduction in both R and the condition number, thus enhancing observability. When $R>R_{safe}$, a smaller δ resulted in a faster decrease in the condition number, as shown in Figure 10d. After R reached $R_{safe}$, the condition number stabilized across different cases. Figure 10c shows that, although it eventually converges to nearly zero in all the scenarios, the localization error was significantly lower with a smaller δ. To exclude the effects of stochastic factors such as Gaussian noise, a Monte Carlo simulation was conducted. The mean and standard deviation (Std) of the root-mean-square error (RMSE) of the localization error over 100 runs are summarized in Table 3, clearly demonstrating that a smaller δ improves target localization performance.

Although a smaller δ implied better observability, it is apparent that δ was not as small as it should be. When δ became too small, the CBF constraints on the UAV became excessively stringent, as illustrated in Figure 10a, resulting in a severely limited feasible area for the UAV. This limitation makes it challenging for UAVs to fulfill certain mission requirements, such as cruising. Therefore, it is crucial to optimize δ to effectively constrain UAV motion based on mission objectives.

### 5.4. Effect of the Initial Flight Path Angle on Trajectory Optimization

To analyze the effect of the initial flight path angle on trajectory optimization, the initial flight path angles θ were divided into six groups: $\theta_{1}=15^{\circ }$, $\theta_{2}=25^{\circ }$, $\theta_{3}=35^{\circ }$, $\;\theta_{4}=55^{\circ }$, $\;\theta_{5}=65^{\circ }$, and $\;\theta_{6}=75^{\circ }$. The optimal solution was chosen by the TCM throughout the process.

**Figure 11:**
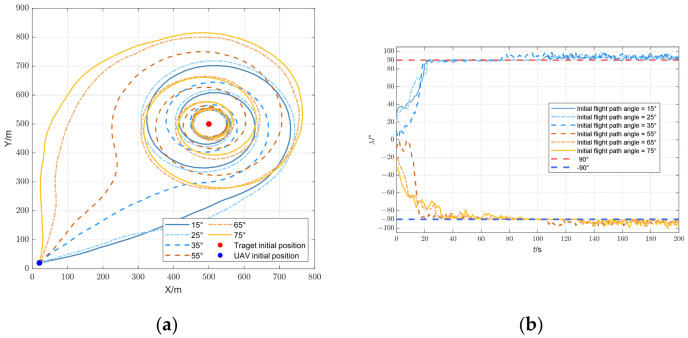
UAV trajectory optimization for different initial flight path angles: (**a**) trajectory of the UAV; (**b**) separation angle.

Figure 11a shows the UAV trajectory for different initial angles. The trajectory was divided into two categories based on a 45° flight path angle: For angles less than 45°, the UAV followed a counter-clockwise circular path around the target with a continuously decreasing radius. Conversely, for angles greater than 45°, the UAV followed a clockwise circular path. Similarly, as shown in Figure 11b, the convergence of the UAV separation angle was categorized into two groups. For initial flight path angles greater than 45°, the separation angle converged to −90°. When the initial flight path angle was less than 45°, it converged to 90°.

When the flight path angle was exactly 45°, the UAV moved directly toward the target, resulting in a separation angle of zero. In this case, the target localization system became unobservable, meaning that the target’s position could not be accurately estimated.

### 5.5. Comparison between the NCMOGWOA and Other Methods

To evaluate the effectiveness of the proposed NCMOGWOA, it was compared with the MOPSOA, MOAOA, and SQP methods [34,35,36]. The same experimental parameters (Table 1) were selected for the NCMOGWOA. The MOPSOA, MOAOA, and SQP parameters used in this study are summarized in Table 4.

**Figure 12:**
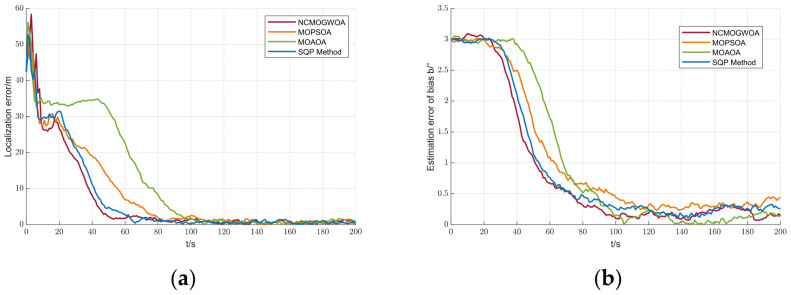
State estimation errors between the NCMOGWOA, MOPSOA, MOAOA, and SQP Methods: (**a**) localization errors; (**b**) estimation error of b.

The results are illustrated in Figure 12. To exclude stochastic effects such as Gaussian noise, a Monte Carlo simulation was conducted. The mean and standard deviation (Std) of the root-mean-square error (RMSE) are summarized in Table 5. As shown in Figure 12 and Table 5, although each method achieved a low localization error, the proposed NCMOGWOA converged more rapidly. It also achieved a lower mean and Std for the target position and bearing bias errors, indicating a superior localization performance.

To assess algorithm performance quantitively, the Generational Distance (GD) and Inverted Generational Distance (IGD) were used to evaluate convergence and comprehensive performance. They are expressed as follows:

(52) $$GD=\frac{\sqrt{\sum_{i=1}^{u}d_{iu}^{2}}}{u}\;\;,\;\;\;IGD=\frac{\sqrt{\sum_{i=1}^{v}d_{iv}^{2}}}{v}$$

where u and v denote the number of obtained Pareto solutions and true Pareto solutions, respectively; $d_{iu}$ denotes the Euclidean distance between the ith obtained Pareto solution and the closest true Pareto solution; and $d_{iv}$ denotes the Euclidean distance between the ith true Pareto solution and the closest true Pareto solution. A smaller value of the GD means better convergence and the lower the value of the IGD is, the better the comprehensive performance of the algorithm.

Table 6 compares the proposed NCMOGWOA with the other methods via the GD and IGD metrics. The NCMOGWOA has slightly lower values, indicating better performance in terms of convergence property. This improvement is due to the effective leader selection strategy, which involves choosing wolves $\partial_{1}$, $\partial_{2}$, and $\partial_{3}$ from the current Pareto front, optimizing the process by leveraging the best results, and exploring diverse solutions across the objective space.

**Figure 13:**
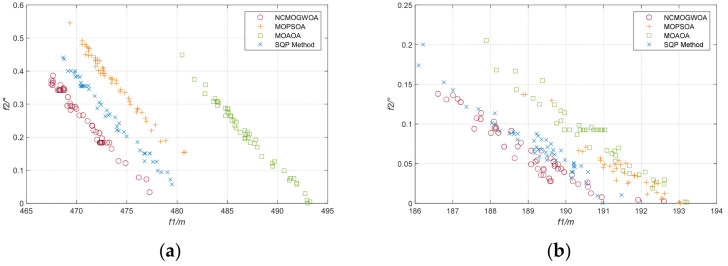
Comparison of the Pareto optimal solution sets over 50 runs: (**a**) t = 10 s; (**b**) t = 50 s.

Figure 13 illustrates the Pareto solutions from different algorithms over 50 runs. Although the Pareto solution sets of the NCMOGWOA, MOPSOA, and SQP were similar when t = 10 s, the NCMOGWOA performed better in both objective functions, initially showing good exploration ability. Additionally, Figure 13a,b shows that the Pareto optimal solutions converged as the simulation time increased, which was consistent with the localization error trend in Figure 12a, where all algorithms eventually optimized the objectives, reducing the localization error to nearly zero.

## 6. Discussion

Most previous studies focused on system observability and maintenance. This study used the condition number of the observability matrix to numerically analyze system observability. We formulated a multi-objective, nonlinear optimization problem for UAV trajectory planning to enhance observability. The proposed NCMOGWOA can efficiently solve this problem.

The simulation demonstrated that optimizing UAV trajectories significantly improved localization system observability and target localization performance. Compared with the other algorithms, NCMOGWOA achieved better performance in both target localization and convergence.

This study focused on a two-dimensional observability matrix. As the dimensions increased, computing the condition number became challenging, limiting its use as a metric. Future work will explore alternative metrics to quantify system observability.

## 7. Conclusions

In this work, we developed a control barrier function inspired by plant phototropism to ensure observability in a target localization system with biased bearing information. This prevents the UAV from entering unobservable areas. By analyzing the condition number of the observability matrix, we identified two influencing factors. We then formulated a multi-objective, nonlinear optimization problem for UAV trajectory planning to enhance system observability. To solve this problem, a penalty function was introduced into the Multi-Objective Gray Wolf Optimization Algorithm (MOGWOA) to manage nonlinear constraints. Simulations confirmed the effectiveness of this trajectory optimization for stationary target localization. Additionally, we analyzed the effects of different CBF attenuation rates and initial flight path angles. Finally, the proposed NCMOGWOA was compared with other algorithms using GD and IGD metrics, which yielded competitive results.

## Figures and Tables

**Table 1:** UAV trajectory optimization parameters for a stationary target.

Parameter	Value
Position for stationary target	(500 m, 500 m)
Initial position for UAV	(20 m, 20 m)
Bearing measurement bias b	$3^{{}^{\circ}}$
Range of UAV speed	[5 m/s, 30 m/s]
Range of UAV turn rate	$\left[-15^{{}^{\circ}}/s,\;\;15^{{}^{\circ}}/s\right]$
Smallest safe distance $R_{safe}$	50 m
Sampling time Δt	0.1 s
Switch distance $R_{switch}$	300 m
Parameters in Equation (35)	α=0.1
Population size $M_{P}$	100
Iteration times $T_{P}$	10
$p_{g},\;p_{f}$	$p_{g}=p_{f}=1000$
Range of δ	[0.01, 0.2]
Grid number $N_{grid}$	30
β in NCMOGWOA	4
γ in NCMOGWOA	2

**Table 2:** RMSEs for the open-loop and closed-loop modes over 100 Monte Carlo experiments.

Mode	Open-Loop Mode	Closed-Loop Mode
Localization error mean, m	14.7082	11.6686
Localization error Std, m	13.4246	11.0246

**Table 3:** RMSEs for different δ values over 100 Monte Carlo experiments.

δ Value	$\delta_{1}=0.06$	$\delta_{2}=0.08$	$\delta_{3}=0.1$
Localization error mean, m	10.0427	10.8554	11.7662
Localization error Std, m	11.0229	11.9279	13.3564

**Table 4:** Simulation parameters for the MOPSOA, MOAOA, and SQP.

Common Parameters			MOPSOA			MOAOA		SQP	
Population Size	Iteration Time	Archive Size	w	$c_{1}$	$c_{2}$	β	γ	α	ρ
100	10	50	0.6	1.2	2	2	2	1	0.5

**Table 5:** RMSE for different algorithms over 100 Monte Carlo experiments.

Algorithm	NCMOGWOA	MOPSOA	MOAOA	SQP Method
Localization error mean, m	13.1646	14.1125	19.4819	13.5077
Localization error Std, m	10.5645	12.6114	15.0696	10.3645
Bearing bias mean, °	1.3446	1.4637	1.5903	1.3921
Bearing bias Std, °	1.1544	1.2938	1.4086	1.1544

**Table 6:** GD and IGD metrics over 100 Monte Carlo experiments.

Algorithm	NCMOGWOA	MOPSOA	MOAOA	SQP Method
GD mean	7.3442	7.9967	8.6906	7.7604
GD Std	5.4062	4.9632	6.2275	5.0342
GD median	6.9088	7.7646	8.8364	7.8621
IGD mean	8.4577	8.8995	9.6554	8.6613
IGD Std	7.8654	6.6877	8.2249	7.2145
IGD median	8.5124	8.6466	9.4336	8.6027

## Data Availability

The data are contained within the article.

## Abbreviations

The following abbreviations are used in this manuscript:

CBF	control barrier function
UAV	Unmanned Aerial Vehicle
LOS	line of sight
GWOA	Gray Wolf Optimization Algorithm
MOGWOA	Multi-Objective Gray Wolf Optimization Algorithm
NCMOGWOA	Nonlinear Constrained Multi-Objective Gray Wolf Optimization Algorithm
TOPSIS	Technique for Order Preference by Similarity to Ideal Solution
CRITIC	Criteria Importance Through Intercriteria Correlation
TCM	TOPSIS and CRITIC Method
EKF	Extended Kalman Filter
NSGA	Nondominated Sorting Genetic Algorithms
SQP	Sequential Quadratic Programming
RMSE	root-mean-square error
Std	standard deviation
MOPSOA	Multi-Objective Particle Swarm Optimization Algorithm
MOAOA	Multi-Objective Arithmetic Optimization Algorithm
SQP	Sequential Quadratic Programming
GD; IGD	Generational Distance; Inverted Generational Distance
Nomenclature	
α, θ, λ	Bearing angle, flight path angle, and separation angle of the UAV
$P_{U}$, $P_{T}$	Position vector of the UAV and target
v, ω	Speed and turn rate of the UAV in m/s and rad/s
R, r	Relative distance and its corresponding vector
$r_{x}$, $r_{y}$	x- and y-axis components of relative distance
h	Target bearing information measured by the UAV
b	Bias in target bearing measurement
X, $\Phi_{t+1,t}$	State vector and state transition matrix from t to t + 1
$L_{f}^{j}h$	The jth order Lie derivative of h with respect to f
$S_{\Delta t}$	The set satisfies control barrier function constraints
$R_{\Delta t}$	The reachable set of states after Δt
δ	Attenuation rate of control barrier function
$C^{*},\;c$	Condition number and its inverse of system observability matrix
$f_{n}$	The nth objective function
$R_{safe}$	The minimum safe distance between the UAV and target
$R_{switch}$	The distance for switching solutions
ρ	Nonlinear constrain penalty function
$Solution_{\;R}$	Pareto optimal solution with smallest value in distance
${Solution}_{TCM}$	Optimal solution selected by the TCM
